# Intergenerational Transmission of Valence Bias Is Moderated by Attachment

**DOI:** 10.1111/desc.70068

**Published:** 2025-08-25

**Authors:** Ashley Humphries, Isabella Peckinpaugh, Grace Kupka, Robert James R. Blair, Nim Tottenham, Maital Neta

**Affiliations:** ^1^ Department of Psychology University of Nebraska‐Lincoln Lincoln Nebraska USA; ^2^ Center for Brain, Biology, and Behavior University of Nebraska‐Lincoln Lincoln Nebraska USA; ^3^ COBRE Center on Sleep, Circadian Rhythms in Child and Adolescent Mental Health Providence Rhode Island USA; ^4^ Region Hovedstadens Psykiatri Børne og Ungdomspsykiatrisk Center Genthofte Hovedstaden Denmark; ^5^ Department of Psychology Columbia University New York New York USA

**Keywords:** attachment, communication, intergenerational transmission, parent–child dyad, valence bias

## Abstract

There are individual differences in how people respond to emotionally ambiguous cues (i.e., valence bias), which have important consequences for mental health, development, and social functioning, yet how these differences develop in childhood and adolescence is unknown. Extensive literature shows that children's cognitive biases, including appraisals in uncertain situations, can be influenced by parents. The current study collected valence bias from parent and child dyads (*n* = 136, children ages 6–17 years, *M* = 10.92, SD = 3.22) using a dual‐valence ambiguity task (i.e., the valence bias task). Using structural equation modeling, we found that a child's valence bias was associated with their parent's valence bias (*β* = 0.283, *p* = 0.005). We also explored the effect of parent–child attachment in three facets (communication, alienation, and trust) on this intergenerational transmission. Communication moderated the relationship between parent and child valence bias, such that higher communication led to a stronger relationship between parent and child valence bias (*β* = 0.03, *p* = 0.04). These findings suggest that one mechanism that supports valence bias development is the parent's bias, and this may be uniquely influenced by the degree of parent–child communication. This tendency to similarly interpret ambiguous stimuli may result from social learning. Specifically, our results support a theory of generalized shared reality where parents and children who have a greater interpersonal connection (i.e., communication) also share a more similar world view (i.e., valence bias). A video abstract of this article can be viewed at https://www.youtube.com/watch?v=xlSDxFDmP7g

**Summary:**

- Valence bias represents variability in appraisals of emotional ambiguity, with some people showing greater negativity, and others more positive.
- There is evidence of intergenerational transmission of valence bias, such that children tend to have a bias that mirrors their parents.
- Transmission was moderated by parent-child attachment, such that children that report greater communication with their parent show a more similar bias to their parent.
- These findings are discussed in the context of theories on development and generalized shared realities.

## 1 Introduction

There are individual differences in how people respond to emotionally ambiguous stimuli, with some people demonstrating a negative bias, even when a positive interpretation is equally valid (valence bias; Neta et al. 2009). For example, a surprise facial expression could be elicited in reaction to a positive event, like a surprise birthday party, or a negative one, like shocking news about a cancer diagnosis. Without context, surprised faces can be interpreted as positive or negative, which allows for wide intersubject variability in valence appraisals (Neta et al. 2009) that are stable across at least a 1‐year period (Harp et al. 2022). This valence bias represents one's tendency to interpret stimuli that have a dual valence ambiguity in a more positive or negative light. This tendency relies first on one's ability to interpret facial expressions, which unfolds gradually over childhood and adolescence, with clearly valenced faces (such as angry or happy faces that are subject to much less intersubject variability) being correctly interpreted in early childhood (Herba and Philips 2004). Ambiguous faces, though, present an interesting circumstance for children—the child must determine if the face is positive or negative, and this decision relies on a valence bias that is still developing. Understanding the factors that contribute to valence bias development is a key goal for the current study.

Previous studies using this task have demonstrated links between valence bias and mental health across the lifespan (Park et al. 2016; Neta and Brock 2021; Clinchard et al. 2024; Harp et al. 2024; Petro et al. 2021). Specifically, a more negative valence bias is associated with greater symptoms of depression and anxiety (Park et al. 2016; Neta and Brock 2021), social disconnection (Harp and Neta 2023; Neta and Brock 2021), stress reactivity (Brown et al. 2017), and daily negative affect (Puccetti et al. 2023). These relationships may arise because a negative valence bias represents the tendency to choose a more negative interpretation when equally valid alternative (including positive) interpretations are available; this fundamental and chronic negativity bias is a key feature of internalizing disorders and related constructs (daily negative affect and social disconnection; Everaert et al. 2017). Notably, current research indicates that childhood and adolescence are periods where developmental changes in valence bias occur, which may have downstream consequences for mental health (Tottenham et al., 2013; Petro et al. 2021).

Given the implications of valence bias, recent work has set out to determine how valence bias develops. One way may be through the parent–child relationship. Theoretical models indicate that the parent–child relationship is a bidirectional dyadic relationship, in which the parent and child mutually influence each other (Pardini 2008). However, in practice, empirical studies often conceptualize and model the parent–child relationship to be unidirectional, where the child is mostly influenced by the parent (Pardini 2008; Paschall et al. 2015). For example, previous work has found evidence of intergenerational transmission of fear and avoidance behaviors, social anxiety, and emotion regulation, with the proposed mechanism being that the parent models these behaviors for the child to observe and learn (Gerull and Rapee 2002; Rosnay et al. 2006). In another experiment, mothers successfully trained their child to have a certain positive or negative bias towards ambiguous stimuli (Remmerswaal et al. 2016). However, in many of these studies, the mechanism of this intergenerational transmission (i.e., influence of parent on child), whether it be shared environmental factors, genetic inheritance, observational learning, or explicit teaching, remains unclear. In the current study, we will investigate whether valence bias can be transmitted intergenerationally. But, as in prior work, we remain agnostic—for now—to the precise mechanism by which this bias is transmitted.

The closeness of the bond between a parent and child is known as their attachment (Armsden and Greenberg 1987), and the quality of this attachment can vary dramatically between parent–child dyads. Notably, parent–child attachment can be quantified using three facets—trust, alienation, and communication (Armsden and Greenberg 1987; Gullone and Robinson 2005). There has been considerable work showing that more secure parent–child attachment (i.e., higher communication and trust, and lower alienation) is positively associated with a child's psychological well‐being, such as self‐esteem and life satisfaction (Armsden and Greenberg 1987; Gullone and Robinson 2005). In general, secure attachments have been associated with positive information processing (Belsky et al. 1996; Feeney and Cassidy 2003; Kirsh and Cassidy 2006; Vannucci et al. 2024). In addition, a meta‐analysis found that a more secure parent–child attachment was associated with a weaker hostile attribution bias (i.e., the tendency to appraise ambiguous actions as hostile; Xu et al. 2024). Therefore, attachment may also shape the way we interpret and react to ambiguous emotional expressions, such as surprise (i.e., valence bias), or shape the way this tendency develops. One study investigated how each facet of attachment predicted emotion regulation use and found that only communication, not trust or alienation, predicted higher cognitive reappraisal and lower suppression use in adolescents (Gresham and Gullone 2012). This is relevant to our work in valence bias given that other studies have shown that a more negative valence bias can be mitigated using cognitive reappraisal (Raio et al. 2021; Brock et al. 2022; Neta 2024), a strategy of emotion regulation that involves reinterpreting negatively valenced stimuli in a less negative or more positive light.

In our daily lives, we often face ambiguous situations, and our response (e.g., valence bias) has been associated with various psychopathologies. However, little is known as to what factors influence valence bias. The current study aims to examine the parent–child relationship to understand one possible influence in shaping valence bias development. We also explored the extent to which facets of parent–child attachment shape the intergenerational transmission of valence bias. We speculate that a more secure relationship between a parent and child may indicate an increased likelihood of the child developing a more similar interpretation bias to the parent. This speculation is in line with theories that parent–child attachment plays a role in childhood socialization, including belief development (Richters and Walters 1991). Given that previous research has demonstrated that the three facets can be independently related to outcomes, we will examine the facets separately.

## 2 Methods

### 2.1 Participants

We recruited 136 parent–child dyads from a midwestern community combining urban and suburban environments as a part of a larger study. Children who completed the study (*N* = 136; 72 males = 52.9%) were aged 6–17 years (*M* = 10.49, SD *=* 3.22) with no history of psychiatric or neurological disorders, nor were they taking psychotropic medications. Parents who completed the study (*n* = 79 unique parents, 87% mothers) were aged 29–53 years (*M* = 41.17, SD *=* 6.37), and they could enroll multiple children in the study. Parent and child demographic information are reported in Table 1.

**TABLE 1:** Sample demographics.

Variable	Levels	*N*	%
Child sex			
	Female	64	47.1
	Male	72	52.9
Child race			
	Asian	1	0.7
	Black or African American	7	5.1
	White	109	80.1
	More than one race	17	12.5
	Missing	2	1.5
Child ethnicity			
	Hispanic or Latino/a	16	11.8
	Not Hispanic or Latino/a	120	88.2
Parent sex			
	Female	69	87.3
	Male	10	12.7
Parent race			
	Asian	1	1.3
	Black or African American	3	3.8
	White	69	87.3
	More than one race	2	2.5
	Missing	4	5.1
Parent ethnicity			
	Hispanic or Latino/a	4	5.1
	Not Hispanic or Latino/a	75	94.9
Parent education			
	Trade/vocational training	3	3.8
	Some college, but no degree	3	3.8
	Associate's degree	7	8.9
	Bachelor's degree	28	35.4
	Master's degree	19	24.1
	Doctorate degree	5	6.3
	Missing	14	17.7

*Note*: Parents could enroll multiple children in the study. Parent demographic information reflects unique parents.

Of our original sample of 136 children and 79 unique parents, valence bias data were excluded for eight children and one parent for inaccurate responses on the valence bias task (described below). Our study, therefore, included complete valence bias data from 128 children and 78 unique parents. Additionally, some questionnaire data were missing for either the child or the parent in 69 of the dyads. However, we used Full‐Information Maximum Likelihood (FIML; see analytic plan) estimation to estimate missing data, allowing us to retain all 136 dyads in analyses. All participants were informed of the procedures, and parents gave written consent and children gave written assent prior to participation. Furthermore, all participants were compensated through monetary payment, and all procedures were approved by the university's Committee for the Protection of Human Subjects.

### 2.2 Measures

**Valence bias task**: Participants viewed images of faces with a positive (happy), negative (angry), and ambiguous valence (surprised) on a black background. The stimulus set included 48 faces: 24 clearly valenced faces (12 positive, 12 negative) and 24 ambiguously valenced faces. Thirty‐four discrete identities were used—14 from the NimStim set (Tottenham et al. 2009) and 20 from the Karolinska Directed Emotional Faces Database (Lundqvist et al. 1998). All face stimuli were White adults to ensure consistency in stimuli and limit the possibility of racial biases impacting interpretation of valence since a majority of our sample was White (cf. Pierce et al. 2025). These stimuli have been used in extensive prior work with this task (Neta et al. 2009; Tottenham et al. 2013). Stimuli were presented in four blocks of trials, alternating between faces and scenes. (Note that the scenes were outside the scope of this report.) Each block included a balance of 12 clear (six positive, six negative) and 12 ambiguous trials, and each face condition contained an equal number of male and female faces.

For each image, children were asked to use the computer mouse to indicate as quickly and as accurately as possible whether the face was “good or bad” and the two response options were in the top corners of the screen (counterbalanced right and left across participants). Parents were given the same instructions, but the response options were “positive or negative,” consistent with prior work in these age‐groups (Neta et al. 2009; Tottenham et al. 2013; Petro et al. 2021). As done previously, valence bias was conceptualized as the percentage of “bad” or “negative” categorizations for the ambiguous stimuli. For example, a child who categorized surprised faces as negative on 80% of surprised trials would have a valence bias of 80%. Clearly valenced stimuli were included as positive and negative anchors and to assess task comprehension; participants who categorized these stimuli below 60% accuracy were excluded, as in extensive prior work (Neta et al. 2009; Petro et al. 2018, 2021; Puccetti et al. 2023). In prior work, we have established high test–retest reliability of valence bias across a period of 6 months, and even 1 year (Harp et al. 2022; Neta et al. 2009).

**Parent–child attachment**: Parent–child attachment was assessed using the Inventory of Parents and Peer Attachment (IPPA; Armsden and Greenberg 1987) for children aged 13–17 years old and the Inventory of Parents and Peer Attachment Revised (IPPA‐R; Gullone and Robinson 2005) for children aged 6–12 years old. The revised measure had updated language to reflect the age of the child (Gullone and Robinson 2005). Items were rated on a 5‐point scale (1 = *almost never true*, 5 = *almost always true*). The IPPA and IPPA‐R contained 28 items that measured a child's attachment with their parent and were represented by the following three subscales: communication, trust, and alienation. The communication subscale assessed the amount and quality of communication between the parent and child, the trust subscale assessed the mutual understanding and respect in the relationship, and the alienation subscale assessed feelings of interpersonal separation and anger between the parent and child (Gullone and Robinson 2005). Higher scores on the communication and trust subscales indicated higher quality attachment. Notably, higher scores for alienation indicated a lack of feelings of alienation, therefore greater attachment. Higher scores on these measures were indexed by greater communication, greater trust, and lower feelings of alienation, which were each indicative of stronger attachment. The IPPA and IPPA‐R items were combined when calculating internal consistency; each subscale (communication, trust, alienation) was acceptable to good (Cronbach's Alpha = 0.72–0.84).

### 2.3 Procedure

After consenting to participate in the study, parents and children first completed the valence bias task in separate rooms, presented on Mousetracker (Freeman and Ambady 2010). After completing the task, parents and children separately filled out surveys related to demographics, and the children filled out the IPPA or IPPA‐R (Armsden and Greenberg 1987; Gullone and Robinson 2005).

### 2.4 Analytic Plan

All analyses were conducted in R studio (Posit Team 2024; version 4.2.0). Correlational analyses used Spearman correlations as Shapiro–Wilks tests indicated non‐normality for all study variables except for Communication (*W* = 0.97, *p* = 0.12; all other ps ≤ 0.01). Then, we used structural equation modeling (SEM) to examine the linear relationship between variables. As opposed to commonly used ordinary least squares (OLS) regression, SEM can handle missing data using FIML and is robust to non‐normal data—which is typical in developmental and social research (Bono et al. 2017). FIML was used in all models and could address the level of missing data within our sample (Enders 2010; Nelson et al. 2021; Neta and Brock 2021).

To examine whether a parent's valence bias predicts a child's valence bias, we used SEM to regress child valence bias onto parent valence bias. The child's age and sex were included as covariates, and exogenous variables were covaried within the model (Model 1). To further support the model, permutation testing was used to determine how robust the observed correlation between a parent's and child's valence bias was compared to a null distribution. The null distribution was generated by shuffling the parent–child relationship such that children were matched with nonparent adults. We then correlated the valence bias between these new dyads and repeated those two steps 1000 times. This process resulted in a null distribution of 1000 correlations, demonstrating the relationship between the valence bias of children and shuffled nonparent adults. To test whether our observed “true” correlation between children and parents was significantly greater than the null distribution, we calculated the proportion of null correlations (between children and nonparent adults) that were stronger than our observed “true” correlation (i.e., we counted the number of null correlations that were equal or greater in strength than the “true” correlation and divided by 1000). To model an exploratory pathway through which the intergenerational transmission of valence bias might be influenced by attachment, we conducted SEM models that built upon Model 1. As the three subscales were predictably correlated with one another (see Results), we included them in three separate models to prevent multicollinearity. Models 2–4 tested the effects of communication, trust, and alienation, respectively. These models were identical to Model 1, except for the removal of sex as a covariate and the addition of the IPPA attachment subscales and their interaction with parent valence bias.

## 3 Results

### 3.1 Valence Bias Ratings

Children and parents accurately rated happy faces as positive (children *M* = 97.20%, SD = 6.55; parents *M* = 99.51%, SD = 1.97) and angry faces as negative (children *M* = 95.24%, SD = 7.86; parents *M* = 98.04%, SD = 5.40). As expected, there was more intersubject variability in their ratings of surprised faces (children *M* = 51.68%, sd = 32.10; parents *M* = 51.02%, sd = 29.65), consistent with prior work (Neta et al. 2009; Tottenham et al. 2013; Petro et al. 2021).

### 3.2 Correlations Between Measures

All three subscales were significantly intercorrelated: communication, trust, and alienation (*rs* = 0.51–0.65, *ps* < 0.001). In addition, parent valence bias was significantly correlated to child valence bias (*r* = 0.26, *p* = 0.011). No other correlations between study variables reached statistical significance (Table 2).

**TABLE 2:** Bivariate correlations.

	Communication	Trust	Alienation	Child VB	Parent VB	Child age	Child sex
Communication	1.00						
Trust	0.65^***^	1.00					
Alienation	0.51^***^	0.63^***^	1.00				
Child VB	0.22	−0.13	0.17	1.00			
Parent VB	0.02	−0.01	0.00	0.26^*^	1.00		
Child age	−0.16	0.18	0.03	0.13	−0.04	1.00	
Child sex	0.25	0.15	−0.01	0.04	0.03	0.06	1.00

*Note*: The alienation subscale measures a *lack* of alienation (see *measures*).

Abbreviation: VB, valence bias calculated from the behavioral task.

* alpha < 0.05, **alpha < 0.01,

*** alpha < 0.001.

### 3.3 Main Model Findings

**FIGURE 1:**
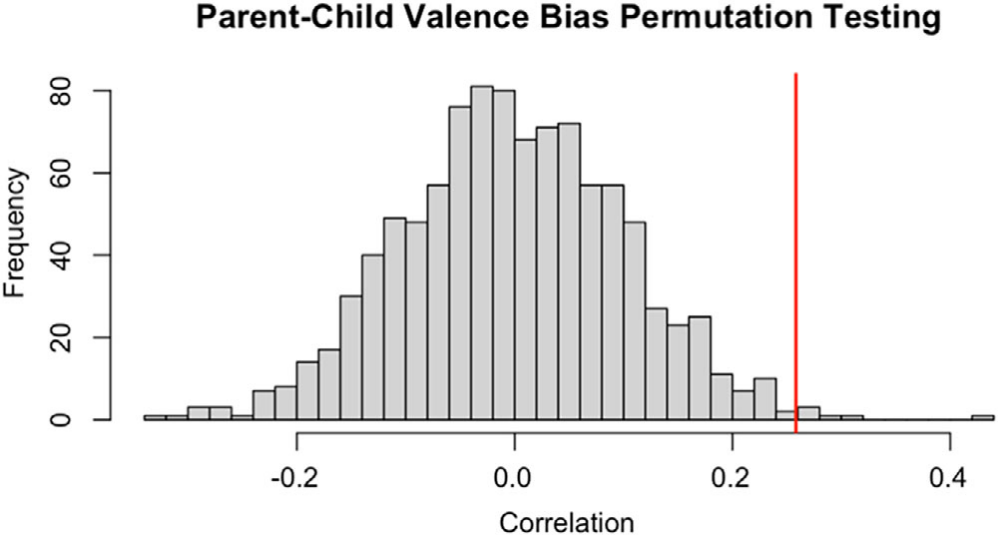
Permutation testing revealed that the true correlation (red line; *r* = 0.26) was significantly higher than the average of 1000 null correlations (*p* = 0.014).

Model 1 examined the intergenerational transmission of valence bias and revealed a significant positive effect of parent valence bias on child valence bias (*β* = 0.283, *p* = 0.005). In addition, there was a nonsignificant, but trending, positive effect of child age (*β* = 0.016, *p* = 0.058), and no significant effect of child sex (*β* = 0.016, *p* = 0.762). The above results held even when sex was removed from the model (see Table S1 in Supporting Materials). Furthermore, although our sample contained a wide age range (6–17 years), there was no significant interaction between child age and parent valence bias (*β* = 0.035, *p* = 0.295)—indicating that the effect of parent valence bias on child valence bias does not significantly differ across age (see Supporting Materials). Permutation testing revealed that, in the null distribution made of nonrelated parent–child dyads, only 14 of the 1000 correlations were as strong as or stronger than the “true” correlation. In other words, our observed correlation (*r* = 0.26) was significantly higher than the null distribution (*p* = 0.014, Figure 1). This indicated that the valence bias between related parents and children was correlated significantly higher than the valence bias between randomly paired parents and children in our sample.

### 3.4 Exploratory Model Findings

**FIGURE 2:**
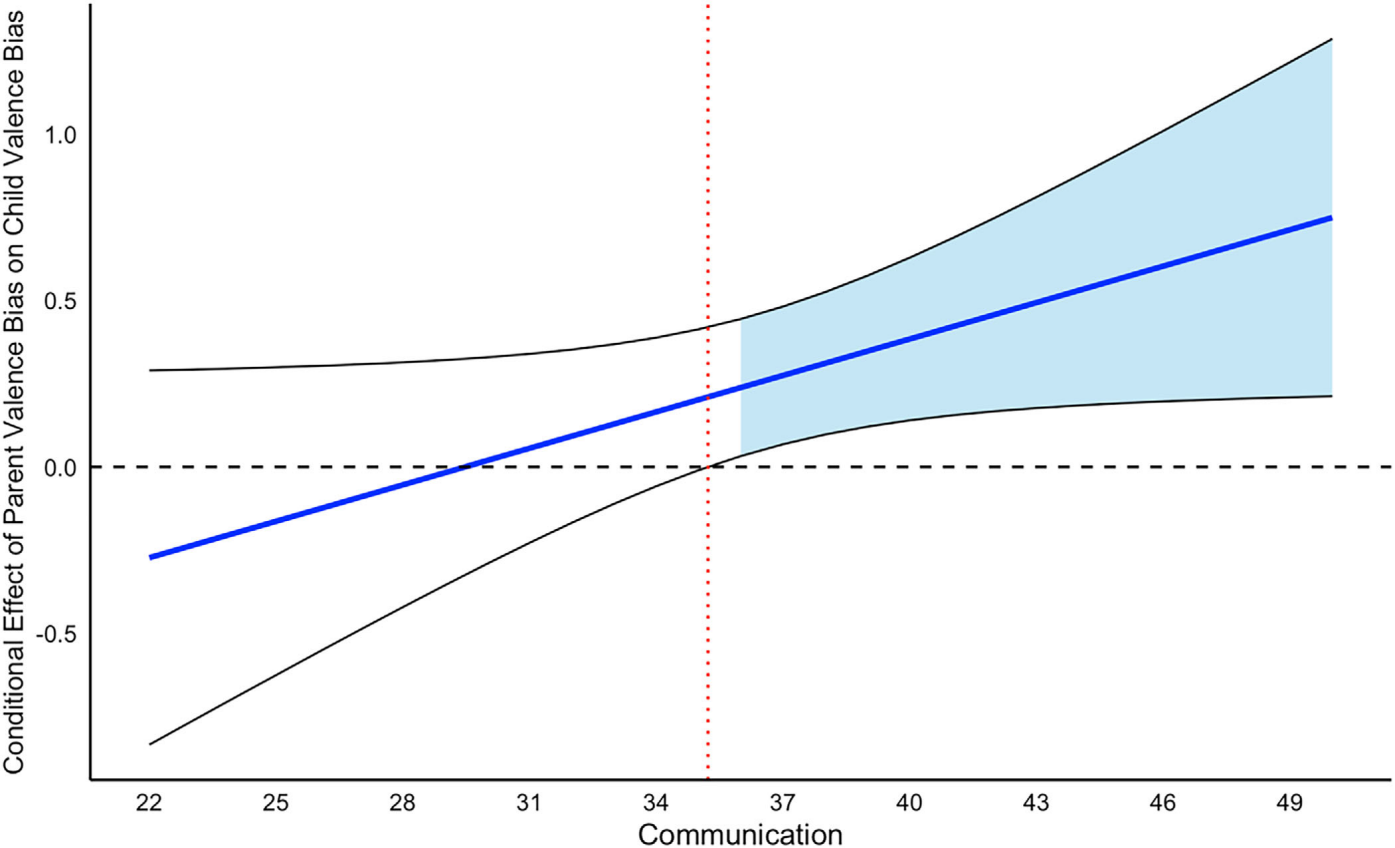
Communication moderates the relationship between parent and child valence bias. The effect of parent valence bias on child valence bias is significant when levels of communication are above the critical point of 35.22 (i.e., communication scores of 36 or above). In such cases, parent valence bias positively predicts child valence bias, indicating a transmission of a similar bias. The black lines represent the 95% confidence intervals for the conditional effect of parent valence bias on child valence bias at that level of communication. The blue shaded area represents all values of communication for which the relationship between parent valence bias and child valence bias is significant at *p* < 0.05.

Models 2–4 iteratively examined the effect of attachment on the intergenerational transmission of valence bias by testing the effects of parent valence bias, parent–child attachment subscales, and their interaction (see Table 2). Model 2 tested effects related to communication and found a significant interaction effect (*β* = 0.037, *p* = 0.047), such that greater communication was associated with a stronger positive relationship between parent and child valence bias (Figure 2). There was also a significant main effect of child age on child valence bias (*β* = 0.020, *p* = 0.021), such that increased age was associated with a more negative valence bias. However, the main effects of parent valence bias (*β* = −1.076, *p* = 0.112) and communication (*β* = −0.007, *p* = 0.555) were not significant.

Model 3 replaced the communication subscale with trust. There was no significant interaction between parent valence bias and trust (*β* = 0.020, *p* = 0.453). There was a significant main effect of child age on child valence bias (*β* = 0.022, *p* = 0.015), such that increased age was again associated with a more negative valence bias, but there were no main effects of parent valence bias (*β* = −0.586, *p* = 0.610) or trust (*β* = −0.001, *p* = 0.960).

Model 4 replaced the trust subscale with alienation. There was a marginally nonsignificant interaction between alienation and parent valence bias (*β* = 0.032, *p* = 0.050), such that lower alienation almost led to a stronger positive relationship between parent and child valence bias. There were no significant main effects of age (*β* = 0.015, *p* = 0.066), parent valence bias (*β* = −0.678, *p* = 0.160), or alienation (*β* = −0.007, *p* = 0.535) (Table 3).

**TABLE 3:** Model results.

				95% CI	
Estimated path	*β*	SE	*p*	LL 2.5%	UL 2.5%
*Model 1*					
Parent VB	0.283	0.102	0.005^**^	0.084	0.482
Age	0.016	0.009	0.058	−0.001	0.033
Sex	0.016	0.054	0.762	−0.090	0.122
*Model 2*					
Communication	−0.007	0.012	0.555	−0.030	−0.016
Parent VB	−1.076	0.677	0.112	−2.403	0.251
Age	0.200	0.009	0.021^*^	0.003	0.036
Communication^*^Parent VB	0.037	0.018	0.047^*^	0.000	0.073
*Model 3*					
Trust	−0.001	0.015	0.960	−0.029	0.028
Parent VB	−0.586	1.149	0.610	−2.837	1.666
Age	0.022	0.009	0.015^*^	0.004	0.039
Trust^*^Parent VB	0.020	0.027	0.453	−0.032	0.073
*Model 4*					
Alienation	−0.007	0.011	0.535	−0.027	0.014
Parent VB	−0.678	0.483	0.160	−1.624	0.268
Age	0.015	0.008	0.066	−0.001	0.031
Alienation^*^Parent VB	0.032	0.016	0.050	0.000	0.064

*Note*: Age and sex refer to the child's age and sex. VB refers to the valence bias score from the behavioral task.

* *p* < 0.05,

** *p* < 0.01, and ***p < 0.001.

## 4 Discussion

We found that children's tendency to interpret ambiguous stimuli as more positive or negative was associated with that of their parents, above other nonfamilial dyads. This suggests the presence of intergenerational transmission of valence bias, whereby children interpret ambiguous stimuli similarly to their parents. Although we did not test a mechanism through which this bias may be transmitted and are therefore unable to rule out other possible pathways, we interpret these findings in a manner consistent with a social learning pathway.

Importantly, the transmission was shaped by children's characterization of communication with their parent, and the transmission did not persist in other randomly paired adult–child dyads. In other words, children who reported lower communication with their parents showed no influence of their parent's valence bias, but increasing levels of communication were associated with a stronger association between parent and child valence bias. Similar effects were evident, though did not reach significance, for alienation. Our findings indicated that the amount and quality of parent–child communication influenced the degree to which the child and parent were similar in their appraisal of ambiguous stimuli. This finding could suggest that the transmission of valence bias from parent to child is influenced by active processes that are shaped via communication pathways (as opposed to being simply modeled by the parent).

Our results are consistent with a robust literature demonstrating that parents dramatically shape the emotional and cognitive development of children. For example, children develop similar cognitive biases and fears as their parents (Gerull and Rapee 2002; Hornik et al. 1987; Remmerswaal et al. 2016; Sorce et al. 1985), and a parent's appraisal of a situation (i.e., reacting with fear versus openness) influences how their child appraises the same situation (Gerull and Rapee 2002; Hornik et al. 1987; Rosnay et al. 2006; Sorce et al. 1985). Early research has shown that people can be influenced to interpret ambiguous stimuli in a positive or negative way depending on training, further indicating that these biases are modifiable (Mathews and Mackintosh 2000; Harp et al. 2022). In the current study, parents and their children completed the task in separate rooms, and children were therefore unaware of how their parents responded during the task. Our findings support the notion that children appraise and react to uncertain situations similarly to their parents. Parents likely model responses to ambiguous or uncertain cues, such that children develop and learn the same approach, and independently use that approach in future situations.

The role of communication between parent and child in facilitating the transmission of valence bias is consistent with social and communication theories on generalized shared realities. A generalized shared reality consists of perceived similarities between oneself and others in attitudes or beliefs about the world, and is critically built and maintained through information sharing (Baek and Parkinson 2022; Baumeister et al. 2018). Establishing a shared reality is important in creating and maintaining social connections—for example, dyads who share similar world views also have greater interpersonal connection (Rossignac‐Millon et al. 2021). This theory of generalized shared realities can be linked to development in light of Bandura's theory of vicarious learning through observation (Bandura 1977). In other words, shared realities expand upon Bandura's theory by emphasizing that the motivation for information sharing is to bolster social connection and build a similar worldview (Baek and Parkinson 2022).

This theory of social connection is relevant in parent–child relationships where strong social connections and information sharing are particularly salient across development. Across a child's development, parents maintain their social connection with their child by sharing information via behavioral (i.e., demonstrations) or verbal (i.e., conversations) approaches. Through this information sharing, not only can their social bond strengthen, but the way that they view the world (i.e., their shared reality) can align more strongly. Our findings were consistent with this theory, given evidence that parents and children with greater information sharing (measured by communication) also shared a more similar world view for ambiguous situations (valence bias). In particular, our results suggest that it may not solely be genetics, or a shared household that facilitates the shared world view, but also stronger parent–child relationships, which are indicated by increased communication. In other words, increased communication between a parent and child not only allows increased opportunities for the child to observe and learn the parent's worldview, but may also signify that the child views the parent as a trustworthy source to model behavior and values.

Interestingly, in our application of this theory, a parent–child generalized shared reality could include a positive or negative valence bias, which will have different consequences. As we have previously mentioned, a more negative valence bias is associated with greater symptoms of depression and anxiety (Park et al. 2016; Neta and Brock 2021), social disconnection (Harp and Neta 2023; Neta and Brock 2021), higher stress reactivity (Brown et al. 2017), and daily negative affect (Puccetti et al. 2023). Our evidence suggested that increased parent–child communication may not always be beneficial, especially in cases where a more negative valence bias is cultivated in a parent and child. Similar effects were evident, though did not reach significance for alienation, whereby decreased levels of alienation between parents and children were related to a stronger association between parent and child valence bias.

Finally, we found a somewhat consistent effect of child age, where increased age within our sample was related to a more negative child valence bias. This effect contradicted previous work that used the same procedure and found children aged 6–9 years old provided more consistently negative ratings of surprised faces than adolescents aged 14–17 (Tottenham et al. 2013). Our findings may be partially explained by our sample demographics as we recruited 58 children aged 6–9 years old, but only 28 adolescents aged 14–17 years old. Therefore, our models benefited from including age as a covariate but may not have been appropriate for examining differences in age‐group surprise ratings.

We recognize four limitations in our design. First, our sample was limited to primarily White, non‐Latino/a participants who were recruited from a midwestern city in the United States. This limits our generalizability as the relationship between parent valence bias, child valence bias, and attachment may present differently across racial/ethnic and cultural identities. Similarly, all stimuli were White, non‐Latino/a faces, further limiting our generalizability. Therefore, our sample demographic and stimuli demographic limitations did not allow us to explore potential racial/ethnic interactions and led to a less representational set of stimuli. Second, we did not collect household income or other measures of socioeconomic status, which are shared within households and may influence valence bias. Third, our data were cross‐sectional, which limits our ability to draw causal inferences. Future studies should examine parent and child valence bias longitudinally to examine the causal influence of parent valence bias on that of the child, and the extent to which communication moderates this causal relationship. And finally, the present data are unable to disentangle whether the intergenerational transmission of valence bias is related to a genetic mechanism, a socioenvironmental mechanism (e.g., modeling, shared environmental factors), or both, and future work could explore these possibilities. It may be the case that although communication is one pathway through which valence bias is transmitted across generations, communication tendencies could be influenced by other factors (e.g., temperament), and in turn, these factors may also independently influence valence bias.

## 5 Conclusions

This study established an intergenerational transmission of valence bias, which was shaped to some extent by parent–child attachment. This shared tendency to interpret ambiguous stimuli as positive or negative may be a result of social learning where the child views how their parent interprets ambiguous situations and then the child adopts this tendency as their own lens. Indeed, greater communication between parents and children supported a stronger transmission of valence bias, suggesting this effect is driven at least in part by social learning. Understanding the development of valence bias in children and adolescents is important as a negative valence bias is associated with increased internalizing symptoms and social disconnection in later development (Petro et al. 2021) and adulthood (Neta and Brock 2021).

## Ethics Statement

All study procedures were approved by the Institutional Review Board; all children provided verbal assent with parent/guardian written consent.

## Conflicts of Interest

The authors declare no conflicts of interest.

## Supporting information




**Supporting Information**: esc70068‐sup‐0001‐SuppMat.docx

## Data Availability

Unfortunately, we did not receive participant consent to upload individual‐level data, but all group‐level datasets generated and/or analyzed in these studies are available from the corresponding author upon request.
