# Supplementary material for: Intergenerational Transmission of Valence Bias Is Moderated by Attachment
Source: Dev Sci. 2025 Aug 25;28(5):e70068. doi: 10.1111/desc.70068 (PMC12376953; doi:10.1111/desc.70068)
Supplement: Supplementary file 1 — Supporting Information: esc70068‐sup‐0001‐SuppMat.docx [file DESC-28-e70068-s001.docx]

Additional Analyses:

As sex was non-significant in Model 1, and removed for further models, we examined whether the original Model 1 results differed when sex was no longer a covariate (Model 1b). Results did not differ.

Further, as there was a wide age range in our sample (6-17 years of age), we ran a test similar to Model 1, but tested interactions with age instead of including it as a covariate (Model 1c). This analysis examined whether the impact of parent valence bias on child valence bias differed as a function of child age. The interaction with age was not significant, indicating that the effect of parent valence bias on child valence bias did not significantly differ across age (*β* = 0.008, *p* = 0.893).

Table S1

Model Results

|  |  |  |  | 95% CI | |
| --- | --- | --- | --- | --- | --- |
| Estimated Path | *β* | SE | *p* | LL 2.5% | UL 2.5% |
| *Model 1b* |  |  |  |  |  |
| Parent VB | 0.283 | 0.102 | 0.005** | 0.084 | 0.483 |
| Age | 0.017 | 0.009 | 0.057 | 0.000 | 0.034 |
| *Model 1c* |  |  |  |  |  |
| Parent VB | -0.070 | 0.363 | 0.847 | -0.782 | 0.642 |
| Age | -0.002 | 0.020 | 0.936 | -0.041 | 0.038 |
| Sex | 0.018 | 0.054 | 0.739 | -0.087 | 0.123 |
| Parent VB*Age | 0.035 | 0.033 | 0.295 | -0.030 | 0.100 |
